# Electroacupuncture Inhibits Cartilage Degeneration in a Rat Knee Osteoarthritis (KOA) Model by Suppressing ADAMTS5 Expression

**DOI:** 10.7759/cureus.73736

**Published:** 2024-11-15

**Authors:** Oyunchimeg Chuluunbat, Hideshi Ikemoto, Takayuki Okumo, Naoki Adachi, Tadashi Hisamitsu, Masataka Sunagawa

**Affiliations:** 1 Department of Physiology, Showa University Graduate School of Medicine, Tokyo, JPN; 2 Department of Orthopedic Surgery, Showa University Fujigaoka Hospital, Yokohama, JPN

**Keywords:** acupoint, adamts5, electro-acupuncture, knee osteoarthritis/koa, synovial tissue

## Abstract

Background

Knee osteoarthritis (KOA) is characterized by cartilage degradation, osteophyte formation, and synovitis. Cartilage degradation in KOA begins with the loss of aggrecan, primarily due to A Disintegrin and Metalloproteinase with Thrombospondin Motif 5 (ADAMTS5), which is produced by chondrocytes and synovial cells and a key target for therapeutic intervention. Current treatments for KOA primarily focus on pain relief, as disease-modifying osteoarthritis drugs (DMOADs) remain unavailable. Electroacupuncture (EA), applying electrical stimulation to acupoints, has been investigated for its potential to alleviate KOA symptoms; however, the specific effects of different acupoint combinations remain unclear. This study investigates the effect of EA on pain and cartilage degeneration in a KOA rat model by examining ADAMTS5 expression in synovial tissue.

Materials and methods

Male Wistar rats were divided into five groups: control, sham-operated, KOA model, KOA treated with EA at ST36 (Zusanli)-LR8 (Ququan) (KOA+LR8), and KOA treated at ST36-Ex-LE2 (Heding) (KOA+Ex-LE2). The DMM (destabilization of the medial meniscus) procedure induced KOA, and EA was applied thrice weekly for four weeks. The rotarod test was used to assess motor coordination, and samples were collected for immunofluorescence, Western blot, and histological analysis. Pain was assessed via c-fos expression in the spinal cord, while Safranin O-Fast Green staining was used to evaluate cartilage degeneration via the Osteoarthritis Research Society International (OARSI) scoring system.

Results

The KOA group post-surgery showed reduced motor coordination, while EA at both ST36-LR8 and ST36-Ex-LE2 enhanced performance (day 28: control: 28.8 ± 0.6, sham: 28.4 ± 3.7, KOA: 19.7 ± 0.9, KOA+LR8: 24.8 ± 1.5, KOA+Ex-LE2: 26.9 ± 1.2). Expression of c-fos, elevated in the KOA group, was significantly suppressed by EA (control: 7.6 ± 0.9, sham: 13.6 ± 2.8, KOA: 24.5 ± 2.1, KOA+LR8: 12.8 ± 0.9, KOA+Ex-LE2: 17.0 ± 1.2). Histologically, KOA rats showed severe cartilage degradation and osteophyte formation, while EA at ST36-Ex-LE2 significantly reduced these changes (control: 0.2 ± 0.1, sham: 0.4 ± 0.2, KOA: 1.8 ± 0.4, KOA+LR8: 1.0 ± 0.2, KOA+Ex-LE2: 0.5 ± 0.2). The ST36-LR8 group also showed improvements, although less pronounced than the ST36-Ex-LE2 group. Western blotting revealed that DMM-induced ADAMTS5 expression was significantly inhibited by EA at ST36-Ex-LE2 but not at ST36-LR8 (control: 1.0 ± 0, sham: 1.2 ± 0.4, KOA: 3.0 ± 0.3, KOA+LR8: 2.1 ± 0.3, KOA+Ex-LE2: 1.4 ± 0.4).

Conclusion

EA at ST36-Ex-LE2 showed a remarkable protective effect on articular cartilage by inhibiting ADAMTS5 expression from synovium, suggesting that it can break the vicious cycle of synovitis and cartilage destruction. In contrast, EA at ST36-LR8 had a moderate effect on cartilage degeneration and ADAMTS5 expression. The difference in efficacy may be due to the anatomical differences between acupoints. ST36-Ex-LE2 coincides with an area rich in synovial fibroblasts and mast cells involved in inflammation and pain. This highlights the importance of acupoint selection to maximize the therapeutic effect of EA. The specificity of this acupoint combination provides a potential strategy for managing KOA and slowing the progression of the disease. Further studies are needed to elucidate the detailed mechanisms behind the effects of EA and explore its potential as an alternative or complementary treatment for KOA.

## Introduction

Knee osteoarthritis (KOA) is a joint disease characterized by cartilage destruction, osteophyte formation, subchondral bone sclerosis, and synovitis. KOA is induced by a complex interaction between various tissues within the joint, including the synovium and cartilage [1-3].

The degradation of articular cartilage, which functions as a shock absorber during joint loading and movement, leads to a loss of joint function and pain, decreasing the patient's quality of life (QOL) [4]. Type II collagen and aggrecan, a large proteoglycan that forms giant hydrated aggregates with hyaluronan in the extracellular matrix, give cartilage the properties necessary to perform its shock-absorbing function [4]. In the degradation of cartilage that leads to the loss of joint function in KOA, the loss of aggrecan occurs before the loss of collagen [4]. The loss of aggrecan is closely associated with A Disintegrin and Metalloproteinase with Thrombospondin Motif 5 (ADAMTS5), a member of the ADAMTS enzyme family [5]. Chondrocytes and synovial cells serve as sources of ADAMTS5 [2,6], and recent research has revealed the involvement of synovitis in KOA [1,2]. Extracellular matrix components (tenascin C, fibronectin, low-molecular-weight hyaluronan, and high mobility group protein B-1 (HMGB-1)), which increase due to mechanical stress from aging or trauma such as meniscus or anterior cruciate ligament injury, bind to pattern recognition receptors (PRRs) such as toll-like receptors (TLR) as damage-associated molecular patterns (DAMPs), activating the innate immune system and inducing synovitis [2,7]. Activated synovial fibroblasts promote the expression of aggrecanases, including ADAMTS5 and matrix metalloproteinases (MMPs), which degrade the extracellular matrix, as well as inflammatory mediators. This leads to the progression of KOA [7]. However, there have been few reports showing that inhibiting ADAMTS5 production from synovial cells can prevent the onset and progression of KOA, and the mechanisms involved are not well understood. Elucidating how to inhibit ADAMTS5 secretion by the synovium will be an important strategy for suppressing the onset and progression of KOA.

The causes of pain in early KOA are diverse, including mechanical stimulation due to unstable movement of the torn meniscus, but the most important is inflammatory pain caused by synovitis [8]. Inflammatory cytokines (IL-1β, TNFα, IL-6) and nerve growth factor (NGF) released by synovitis increase the excitability of nociceptor receptors and ion channels in sensory neurons, leading to a lowered threshold for noxious stimuli to the joint (peripheral sensitization) [2,8]. Furthermore, persistent noxious stimuli to the peripheral nerves induce sensitization of spinal dorsal horn neurons (central sensitization), leading to allodynia, where non-noxious stimuli are perceived as noxious stimuli, and hyperalgesia, in which noxious stimuli are perceived more strongly than usual [8]. *c-fos*, an immediate early gene, is expressed in neurons in response to noxious stimuli, and *c-fos* has been used as a marker of pain expression [9]. In fact, *c-fos* expression is also increased in the spinal dorsal horn of KOA model animals [10].

Electroacupuncture (EA) is a treatment in which electrodes are attached to needles inserted into the body, and a weak electric current is passed through them. In traditional Chinese medicine, there are 14 meridians, which are pathways through which Qi (life energy) flows throughout the body [11]. Along these meridians, there are 361 classical acupoints and 48 extra acupoints that are not located on the meridians but have their own specific indications and therapeutic effects [11]. EA applied to these acupoints has been proven effective for various diseases [12], and there are many reports on the effectiveness of EA for knee joint diseases [13,14]. In acupuncture treatment, the selection of acupoints for needle insertion is important, and it has been reported that inserting a needle into an inappropriate acupoint does not provide sufficient therapeutic effect [11,14]. This is called "acupoint specificity," but there is still a lack of research to clarify the scientific evidence [11].

Regarding EA treatment for KOA, studies using the classical acupoints ST36 (Zusanli) [13,14] and LR8 (Ququan) [13] and the extra acupoint Ex-LE2 (Heding) [13,14] have reported improvements in pain and joint function in KOA. Sun et al. reported that these three acupoints are frequently used in the treatment of KOA [14]. While acupuncture treatment at a single classic acupoint is effective, combining a classic acupoint with an extra acupoint is more effective [15]. However, the detailed mechanisms remain unclear. Furthermore, although EA has demonstrated disease-modifying osteoarthritis drug (DMOAD)-like effects by suppressing the expression of MMPs and aggrecanases from chondrocytes [16], there are no reports showing that EA inhibits articular cartilage degeneration via suppressing the expression of ADAMTS5 from synovial fibroblasts.

Based on these backgrounds, we hypothesized that EA treatment reduces pain and cartilage degeneration through the regulation of ADAMTS5. To clarify this hypothesis, we created a KOA model rat and aimed to determine whether EA suppresses ADAMTS5 expression in synovial tissue, thereby inhibiting articular cartilage degeneration. Furthermore, we aimed to investigate whether different combinations of acupoints produce distinct therapeutic effects.

## Materials and methods

Male Wistar rats (12 weeks old) were purchased from Sankyo Labo Service Corporation, Inc. (Tokyo, Japan). Animals were housed in standard plastic cages (for a group habitat, W 24 cm × L 40 cm × H 20 cm) and were kept in our animal facility at 25°C ± 2°C and 55% ± 5% humidity, with a light/dark cycle of 12 hours during the experiment. Food (CLEA Japan, CE-2, Tokyo, Japan) and water were provided ad libitum. The experiments were performed following the guidelines of the Committee of Animal Care and Welfare of Showa University and the Animal Research: Reporting of In Vivo Experiments (ARRIVE) guidelines for the reporting of animal studies. The Committee of Animal Care and Welfare of Showa University approved all experimental procedures (certificate number: 05023; date of approval: April 1, 2023). All efforts were made to minimize animal suffering and use the minimum number of animals necessary to conduct this study with reliable results. The present study used 115 rats that were randomized and used only once. All observers who scored behavior and performed data analysis were blinded to treatment allocation.

Animals were assigned to the following five groups: (1) a control group, (2) a sham-operated group (sham), (3) a DMM (destabilization of the medial meniscus)-operated group (KOA), (4) a DMM-operated group treated with EA at ST36-LR8 (KOA + LR8), and (5) DMM-operated group treated with EA at ST36-Ex-LE2 (KOA + Ex-LE2). After DMM surgery, EA was administered for 30 minutes three times a week for four weeks [17]. The rotarod test was performed on 30 rats before DMM surgery and 7, 14, 21, and 28 days after surgery [17].

**Figure 1:**
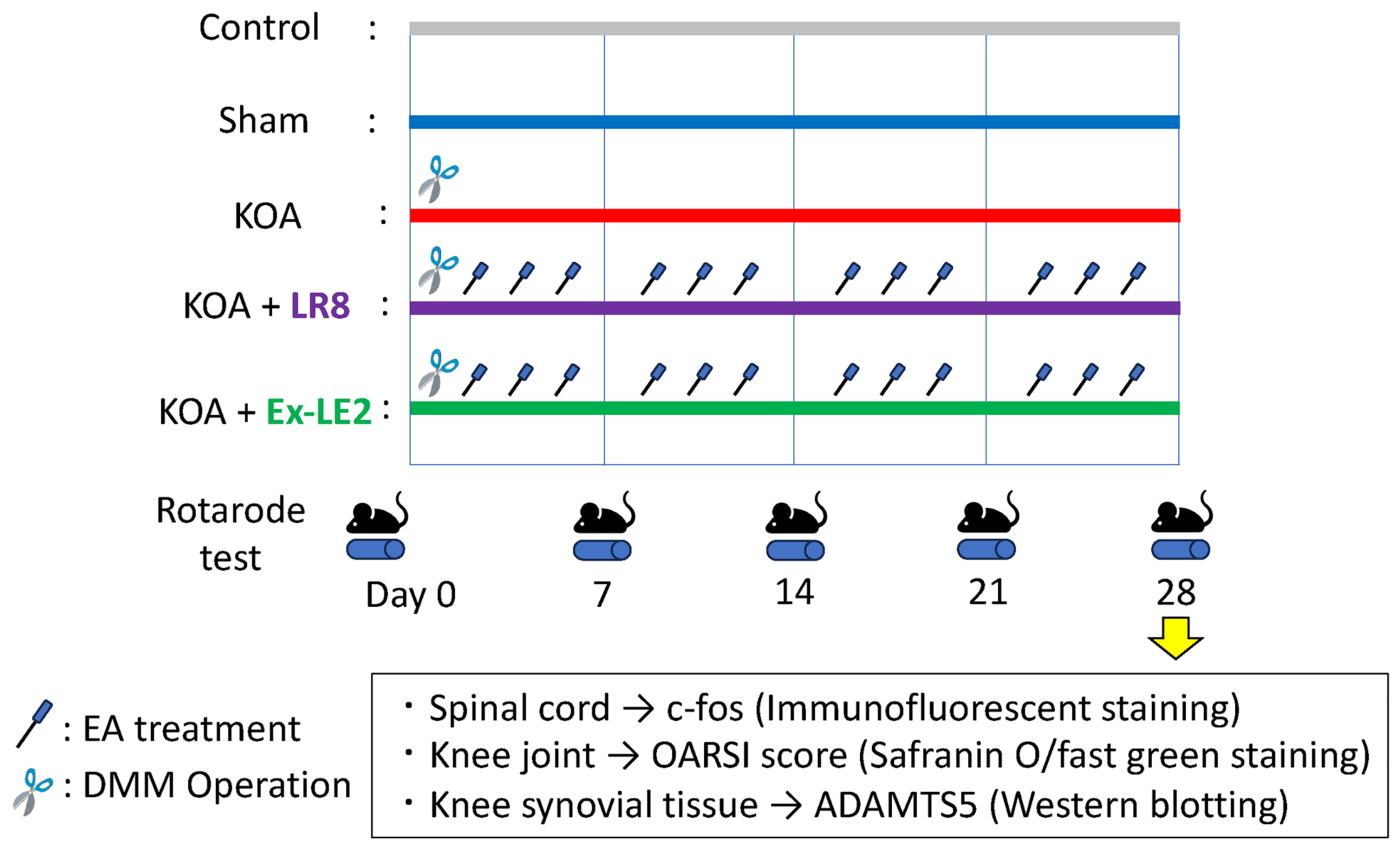
Experimental protocol. Animals were assigned to the following five groups: (1) a control group, (2) a sham-operated group (sham), (3) a DMM-operated group (KOA), (4) a DMM-operated group treated with EA at ST36-LR8 (KOA + LR8), and (5) a DMM-operated group treated with EA at ST36-Ex-LE2 (KOA + Ex-LE2). EA was administered three times a week, for 30 minutes each time, for four weeks after DMM surgery. The rotarod test was performed on 30 rats before surgery and on days 7, 14, 21, and 28 after surgery. Samples were collected after four weeks. EA, Electroacupuncture; DMM, Destabilization of the medial meniscus; KOA, Knee osteoarthritis; ST36, Zusanli; LR8, Ququan; Ex-LE2, Heding; OARSI, Osteoarthritis Research Society International

Four weeks after treatment with EA, samples were collected from the sacrificed animals. The rats were anesthetized by an intraperitoneal administration of a combination of three anesthetics: medetomidine hydrochloride, 0.3 mg/kg (Domitor; Nippon Zenyaku Kogyo Co., Ltd., Fukushima, Japan); midazolam, 4.0 mg/kg (Sandoz; Sandoz K.K. Tokyo, Japan); and butorphanol, 5.0 mg/kg (Vetorphale; Meiji Seika Pharma Co., Ltd., Tokyo, Japan). The 60 rats were intracardially perfused with phosphate-buffered saline (PBS) at pH 7.4 and then with 4% paraformaldehyde in 0.1 M PBS, and the spinal cords (L3) [18] and right knee joints were removed and stored for 48 hours in 4% paraformaldehyde solution. The spinal cord was subjected to immunofluorescent staining, and the knee joint was subjected to Safranin O-Fast Green staining. The rats were euthanized after deeply anesthetizing with a combination of anesthetics, and synovial tissues of the right knee joint were immediately removed and frozen in liquid nitrogen. Knee synovial tissue was used for Western blot analysis (Figure 1).

Meniscal dysfunction resulting from meniscal tear has been identified as one of the key contributors to post-traumatic KOA, and a similar pathological process can be replicated using the surgical procedure of DMM in a rodent animal model. DMM and sham surgeries were conducted on the right knee under isoflurane (Fujifilm Wako Pure Chemical Corp., Osaka, Japan) inhalation general anesthesia, as described in previous studies [17]. Briefly, a 2-cm longitudinal skin was made, followed by a medial parapatellar intervention performed to observe the articular joint space and medial meniscus. The medial meniscotibial ligament (MMTL) was identified behind the patellar tendon. Both the MMTL and the medial meniscotibial joint capsule were transected horizontally to cause meniscal destabilization. In the sham group, only the medial joint was opened.

**Figure 2:**
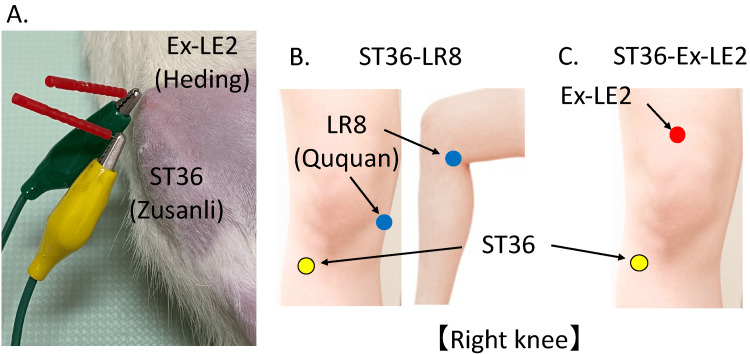
Acupoints for electroacupuncture. Location of the acupoints used in this experiment: (A) actual image of EA administered to rats (combination of ST36 and Ex-LE2); (B) ST36 (Zusanli) and LR8 (Ququan) in humans; (C) ST36 (Zusanli) and Ex-LE2 (Heding) in humans. EA, Electroacupuncture

Four weeks following the DMM surgery, EA treatment was administered using a stainless-steel needle (0.16 mm in diameter, 15 mm in length; Seirin Co., Shizuoka, Japan) and an electrostimulator (Ohm Palser LFP-4000A; Zen Iryoki Co., Fukuoka Japan). EA was applied by delivering a square-wave pulse current between the two needles, and the parameters of electrical stimulation were as follows: pulse duration of 0.1 ms, intensity of 15 mA, and frequency of 2 Hz for 30 minutes [19]. In this experiment, EA was used at three acupoints: ST36 [13,14], LR8 [13], and EX-LE2 [13,14] (Figure 2). The ST36 is located below the knee, on the tibialis anterior muscle, along the stomach meridian. LR8 is located in the depression of the anterior border of the insertions of semimembranosus muscle and semitendinosus muscle. When the knee is flexed, the point is at the medial end of the transverse popliteal crease‎. EX-LE2 is one of the extra acupoints and is located in the depression at the midpoint of the superior border of the patella [20]. We created two pairs of these three acupoints, the ST36-LR8 pair and the ST36-Ex-LE2 pair, and compared the effects of EA via normal acupoints and extra acupoints that are considered to have specific effects on the knee.

The rotarod test was used to assess locomotive function and pain sensitization [17,21]. In this study, an automated accelerating rotarod apparatus (lane width: 75 mm; a rod diameter: 60 mm) (LE8305, Panlab Harvard Apparatus, Barcelona, Spain) was employed. The rats were trained for five minutes daily for two days to adapt to the experiment. The rotating drum was accelerated from 5 to 40 rpm over 30 seconds [17]. The time the rats spent on the gradually accelerating rod was then measured and compared between groups.

The L3 spinal cord samples preserved in 4% paraformaldehyde solution were immersed in 20% sucrose solution for 48 hours [18]. The tissues were subsequently embedded and frozen in Tissue-Tek optimum cutting temperature (OCT) compound (Tissue-Tek; Sakura Finetek, Tokyo, Japan) and frozen at −80°C. The OCT-embedded spinal cords were cut into 20-μm slices by a cryostat (CM1860; Leica Biosystems, Nussloch, Germany). After the sections were rinsed with PBS three times, for blocking and permeabilization, they were incubated with 10% goat serum containing 0.5% triton-X (Sigma-Aldrich Japan Co., Tokyo, Japan) for 2 hours. The sections were then incubated with rabbit anti-*c-fos* antibody (1:500, #2,250, Cell Signaling Technology, Danvers, MA, USA) and mouse anti-NeuN, a specific marker for neurons, antibody (1:1000, #MAB377; MilliporeSigma, Burlington, MA, USA) at 4°C overnight, and then incubated for 2 hours with secondary antibodies with fluorescence tags, anti-mouse Alexa Fluor 555 (1:1000, #A31570; Thermo Fisher Scientific, Waltham, MA, USA) and anti-rabbit Alexa Fluor 488 (1:1000, #A21206; Thermo Fisher Scientific). After a three-time washing, the nuclei were stained with DAPI (4', 6-diamidino-2-phenylindole, 1:3000; Thermo Fisher Scientific) for 10 minutes. A confocal laser scanning fluorescence microscope (FV1000D, Olympus, Tokyo, Japan) was used to capture images of the samples. Further, a third person, who is not engaged in the staining process, counted cells, with co-localization of *c-fos*, NeuN, and DAPI in the same area of laminae I-V as *c-fos*-positive cells. The mean value was calculated using three sequential sections from each rat.

Knee joint samples fixed in paraformaldehyde were subjected to calcium removal for four weeks in 20% ethylenediaminetetraacetic acid disodium salt dihydrate (EDTA) (pH 7.4). The knee joint tissues were embedded in paraffin and sectioned into 4-μm-thick slices. Serial sections were taken from the medial and lateral compartments with 200-μm intervals. Selected sections were deparaffinized using xylene, rehydrated through washes with a graded series of ethanol, and stained with 0.1% Safranin-O and 0.05 % Fast Green. The Osteoarthritis Research Society International (OARSI) score was used to evaluate the degree of articulatio genus cartilage degeneration on the medial and lateral tibial plateau joint [17]. Cartilage degeneration was scored from 0 to 15 points, subchondral bone destruction from 0 to 5 points, osteophyte formation from 0 to 4 points, and the total score ranged from 0 to 24 points. Lower scores indicate less joint degeneration. The mean value of the three slices was taken as the OARSI score for each knee [17]. Furthermore, the scores were assessed by a person not involved in the staining procedure.

The knee synovial tissues were homogenized with lysis buffer containing 1% sodium dodecyl sulfate (SDS), 20 mM Tris-HCl (pH 7.4), 5 mM EDTA (pH 8.0), 10 mM sodium fluoride, 2 mM sodium orthovanadate, 0.5 mM phenylarsine oxide, and 1 mM phenylmethylsulfonyl fluoride. The homogenate was then centrifuged at 10,000 g for 30 minutes at room temperature, and the supernatant was used. After standardization of the concentration of all samples using the BCA protein assay kit (Thermo Fisher Scientific), 10 μg samples were subjected to sodium dodecyl sulfate polyacrylamide gel electrophoresis (SDS-PAGE, 10% SDS) and transferred to a polyvinylidene difluoride membrane. Blocking of the membranes was performed with 5% (w/v) BSA (#011-21271; Fujifilm Wako Pure Chemical) for 1 hour at room temperature. After incubation with primary antibodies overnight at 4°C, rabbit anti-ADAMTS5 antibody (1:500, #ab41037; Abcam, Cambridge, UK) and beta-actin (1:1000, #4970; Cell Signaling Technology, Billerica, MA), the membrane was washed with tris-buffered saline buffer three times and incubated with the goat anti-rabbit secondary antibody conjugated with horseradish peroxidase (1:1000, #611-1302; Rockland Immunochemicals, Gilbertsville, PA) for 1 hour at room temperature. Beta-actin was used as a loading control. Chemiluminescence signals were visualized with Pierce™ ECL Western blotting substrate (Thermo Fisher Scientific) and captured with a charged-coupled device camera system (Ez-Capture MG; Atto Co., Tokyo, Japan). The immunoreactivity of each band was quantiﬁed using Lane & Spot Analyzer software (Atto Co., Amherst, NY, USA).

All data of experiments were presented as mean ± standard error of the mean. One-way analysis of variance (ANOVA) was used to evaluate the statistical significance of the differences among groups. Tukey's test via IBM SPSS Statistics for Windows, Version 25 (Released 2017; IBM Corp., Armonk, New York) was used for post-hoc comparisons between the groups. All p-values of < 0.05 indicated statistical significance. F(df1, df2) = F represents the result of an ANOVA, where F is the F-statistic, df1 is the degrees of freedom in the numerator (between-group degrees of freedom), and df2 is the degrees of freedom in the denominator (within-group degrees of freedom).

## Results

**Figure 3:**
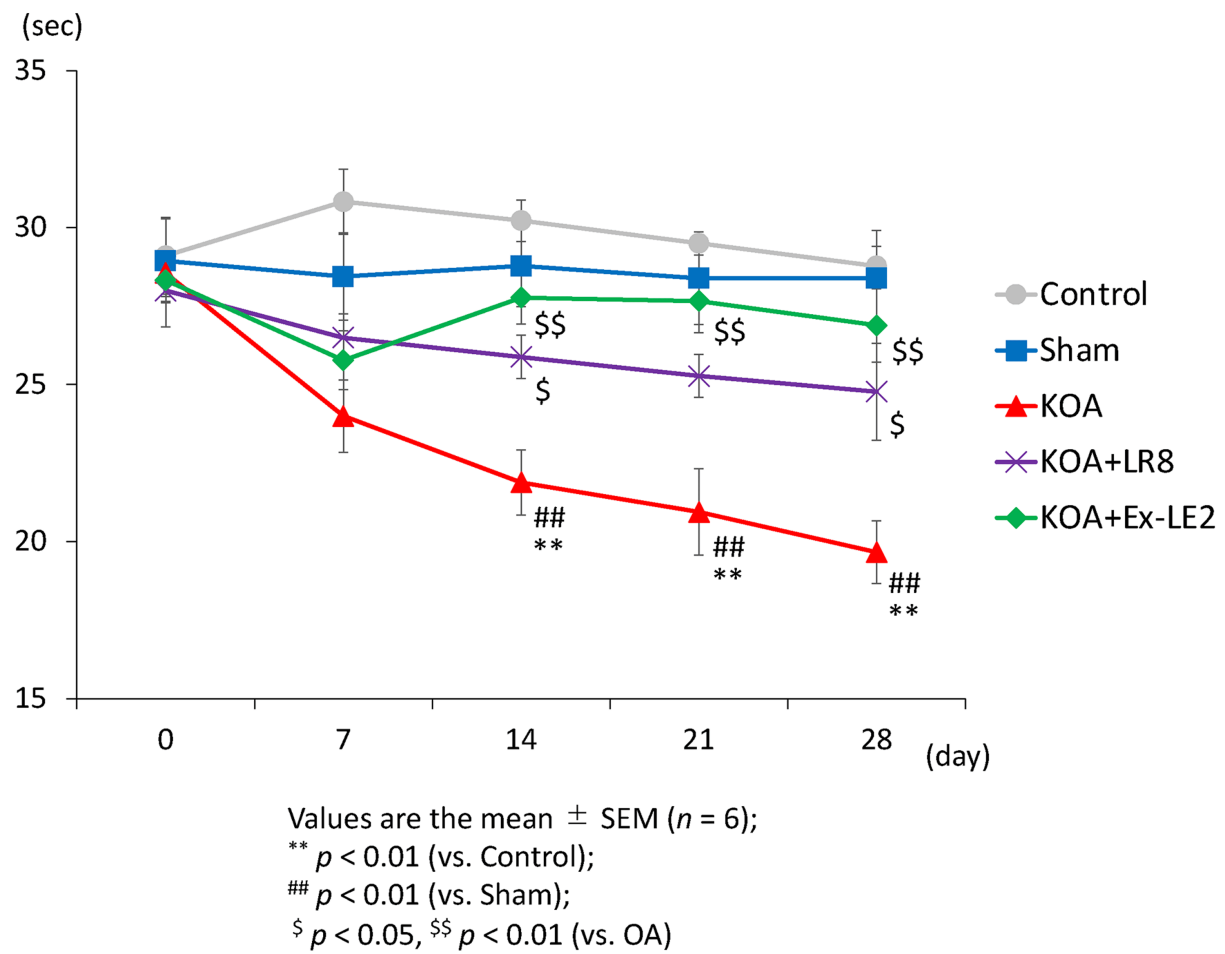
EA at ST36-LR8 and ST36-Ex-LE2 enhances motor function in KOA rats. Electroacupuncture alleviated the decrease in motor coordination. The rotarod test was performed before surgery and on days 7, 14, 21, and 28 post-surgery. The time spent on the rotarod apparatus is shown. All data represent mean ± SEM (n = 6). ^**^ P < 0.01 versus the control group, ^## ^P < 0.01 versus the sham group, ^$^ P < 0.05, ^$$^ P < 0.01 versus the KOA group based on the ANOVA followed by the Tukey's test. KOA, Knee osteoarthritis; ST36, Zusanli; LR8, Ququan; Ex-LE2, Heding

The rotarod test was conducted to evaluate the motor coordination ability of the rats. As previously reported, there were no significant differences between groups on day 7 post-operation, and the KOA group treated with DMM showed a reduced duration of stay on the rod on days 14, 21, and 28 post-surgery compared to the control and sham groups (day 14 F(4,25) = 12.07, p < 0.01, day 21 F(4,25) = 10.22, p < 0.01, day 28 F(4,25) = 9.29, p < 0.01) (Figure 3) [17]. EA at both ST36-LR8 and ST36-Ex-LE2 significantly inhibited the reduction in the duration of stay on the rod observed in the KOA group from day 14 to day 28, except on day 21 in the ST36-LR8 group (ST36-LR8 group: day 14 F(4,25) = 12.07, p < 0.05, day 21 F(4,25) = 10.22, p = 0.06, day 28 F(4,25) = 9.29, p < 0.05. ST36-Ex-LE2 group: day 14 F(4,25) = 12.07, p < 0.01, day 21 F(4,25) = 10.22, p < 0.01, day 28 F(4,25) = 9.29, p < 0.01) (Figure 3). There was no significant difference between the ST36-LR8 and ST36-Ex-LE2 groups (Figure 3).

**Figure 4:**
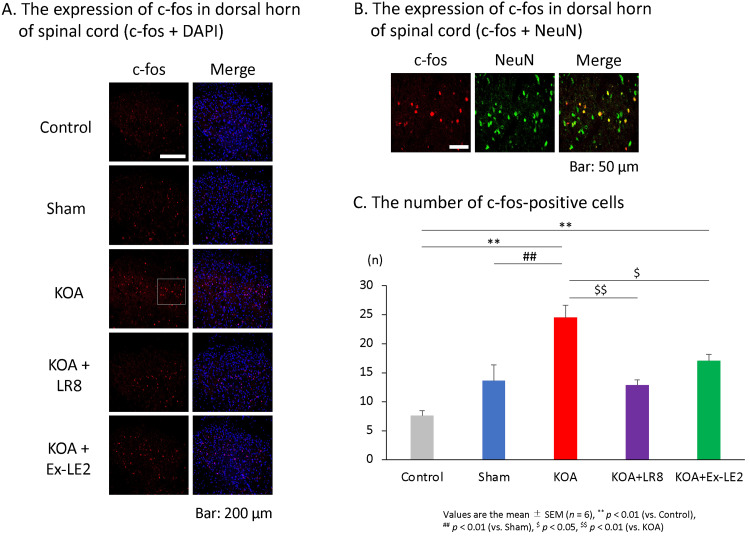
EA decreased c-fos-positive neurons in the dorsal horn of the spinal cord in KOA rats. Electroacupuncture alleviated knee joint pain in the KOA rat model. (A) Immunohistochemical analysis of *c-fos* in the dorsal horn of the spinal cord. Images of *c-fos* immunoreactivity (red) in the dorsal horn of the spinal cord (L3) (white bar = 100 μm). Blue is DAPI. (B) Images showing overlap of *c-fos* and NeuN. An enlarged image of the white square area. (C) Number of *c-fos*-positive cells in the right dorsal horn. Cells where c-fos and DAPI overlapped were counted as *c-fos*-positive cells. All data represent mean ± SEM (n = 6). ^**^ P < 0.01 versus the control group, ^##^ P < 0.01 versus the sham group, ^$^ P < 0.05, ^$$^ P < 0.01 versus the KOA group based on the ANOVA followed by the Tukey's test. KOA, Knee osteoarthritis; ST36, Zusanli; LR8, Ququan; Ex-LE2, Heding

The decrease in the time spent on the rod was likely due to knee pain and decreased muscle strength of the leg that grasped the rod [21]. Therefore, we examined the expression level of *c-fos*, a marker of pain, in the dorsal horn of the spinal cord to visualize the degree of pain sensation in each group. The *c-fos* expression in the dorsal horn of the spinal cord was determined on day 28. The *c-fos* immunoreactivity in the KOA group increased in the right dorsal horn after DMM operation compared to the control and sham group, which was suppressed in both the ST36-LR8 and ST36-Ex-LE2 groups (Figure 4A). In addition, since the expression of *c-fos* overlapped with that of NeuN, we confirmed that *c-fos* was expressed in neurons (Figure 4B). The number of *c-fos*-positive cells in the right dorsal horn also increased by DMM operation compared to the control and sham groups (F(4,25) = 12.80, p < 0.01), but this increase in the KOA group was inhibited by EA (ST36-LR8 group: F(4,25) = 12.80, p < 0.01. ST36-Ex-LE2 group: F(4,25) = 12.80, p < 0.05) (Figures 4A, 4C). In parallel with the changes observed in the motor coordination levels, EA significantly inhibited the KOA-induced *c-fos* immunoreactivity (Figures 4A, 4C). There was no significant difference between the ST36-LR8 and ST36-Ex-LE2 groups (Figures 4A, 4C). These data indicate that EA suppresses pain and does not induce a decrease in mobility.

**Figure 5:**
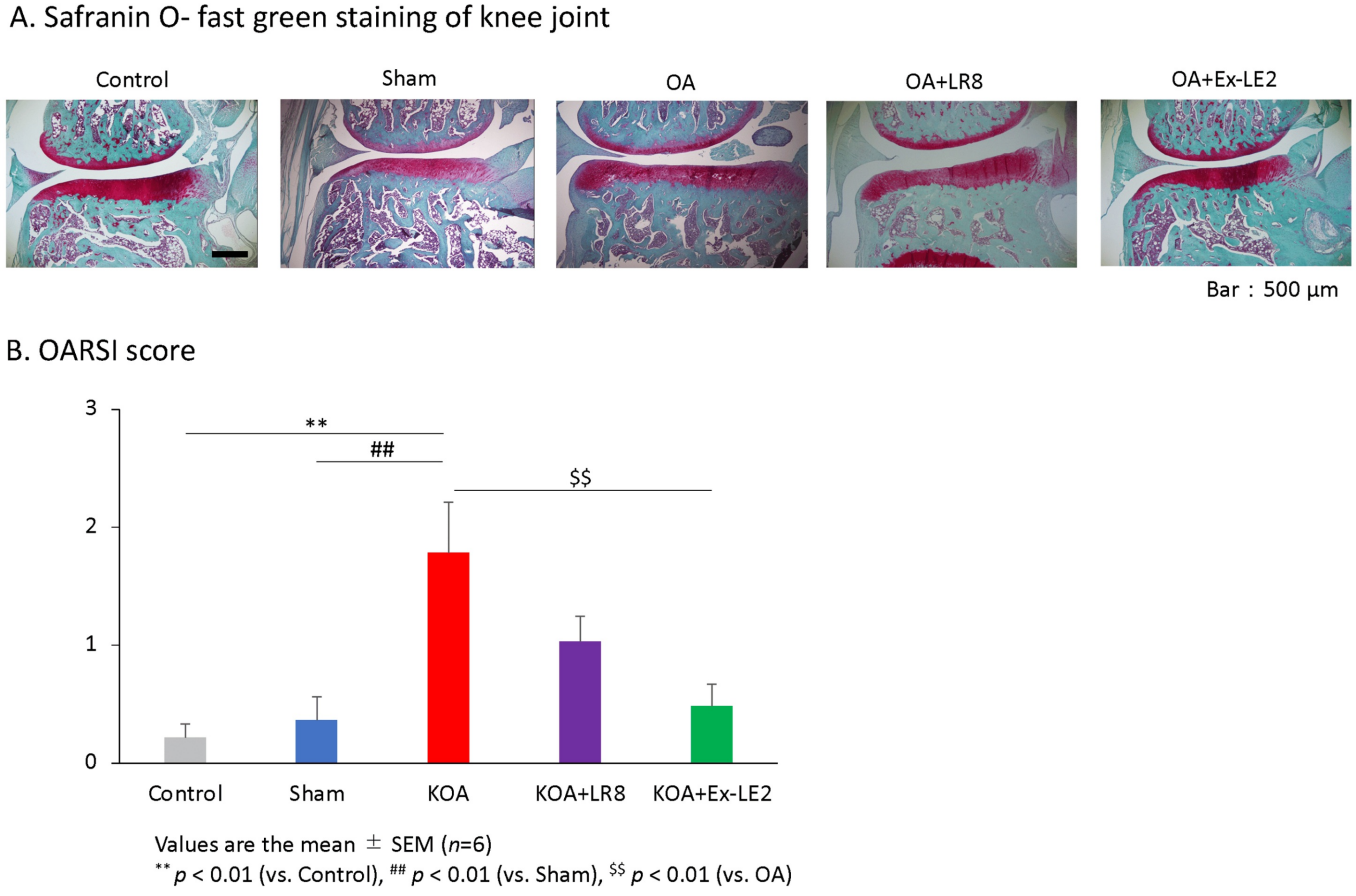
Comparative analysis of EA on cartilage degeneration in KOA rats. Electroacupuncture at ST36 and Ex-LE2 ameliorated KOA development in the KOA rat model. (A) Representative Safranin O-Fast Green staining of the knee joint in each group on day 28 post-surgery. (B) OARSI scores of the knee joints in each group. All data represent mean ± SEM (n = 6). ^**^ P < 0.01 versus the control group, ^##^ P < 0.01 versus the sham group, ^$$^ P < 0.01 versus the KOA group based on the ANOVA followed by the Tukey's test. KOA, Knee osteoarthritis; OARSI, Osteoarthritis Research Society International; ST36, Zusanli; LR8, Ququan; Ex-LE2, Heding

Safranin O-Fast Green staining of the rats' paraffinized knee joint slices revealed articular cartilage degradation, subchondral bone destruction, and osteophyte formation in the KOA group (Figure 5A). In the OA group, degeneration of articular cartilage and formation of osteophytes were observed, but these changes were hardly found in the ST36-Ex-LE2 group. In the ST36-LR8 group, degeneration of articular cartilage and osteophyte formation were observed in some animals (Figure 5A). The severity of OA was evaluated using the OARSI score based on these images. The mean OARSI scores were 0.2 ± 0.1 in the control group, 0.4 ± 0.2 in the sham group, 1.8 ± 0.4 in the OA group, 1.0 ± 0.2 in the ST36-LR8 group, and 0.5 ± 0.2 in the ST36-Ex-LE2 group (Figure 5B). The score was significantly higher in the OA group compared to the control and sham groups (F(4,25) = 6.49, p < 0.01) (Figure 5B). However, this increase was significantly suppressed in the ST36-Ex-LE2 group (F(4,25) = 6.49, p < 0.01), while the same result was not observed in the ST36-LR8 group (F(4,25) = 6.49, p = 0.25) (Figure 5B). On the other hand, there was no significant difference between the ST36-LR8 group and the control (F(4,25) = 6.49, p < 0.18) or sham (F(4,25) = 6.49, p = 0.36) groups. In other words, EA at ST36-LR8 has an effect of preventing cartilage degeneration, but the effect is not as strong as that of ST36-Ex-LE2 (Figure 5B).

**Figure 6:**
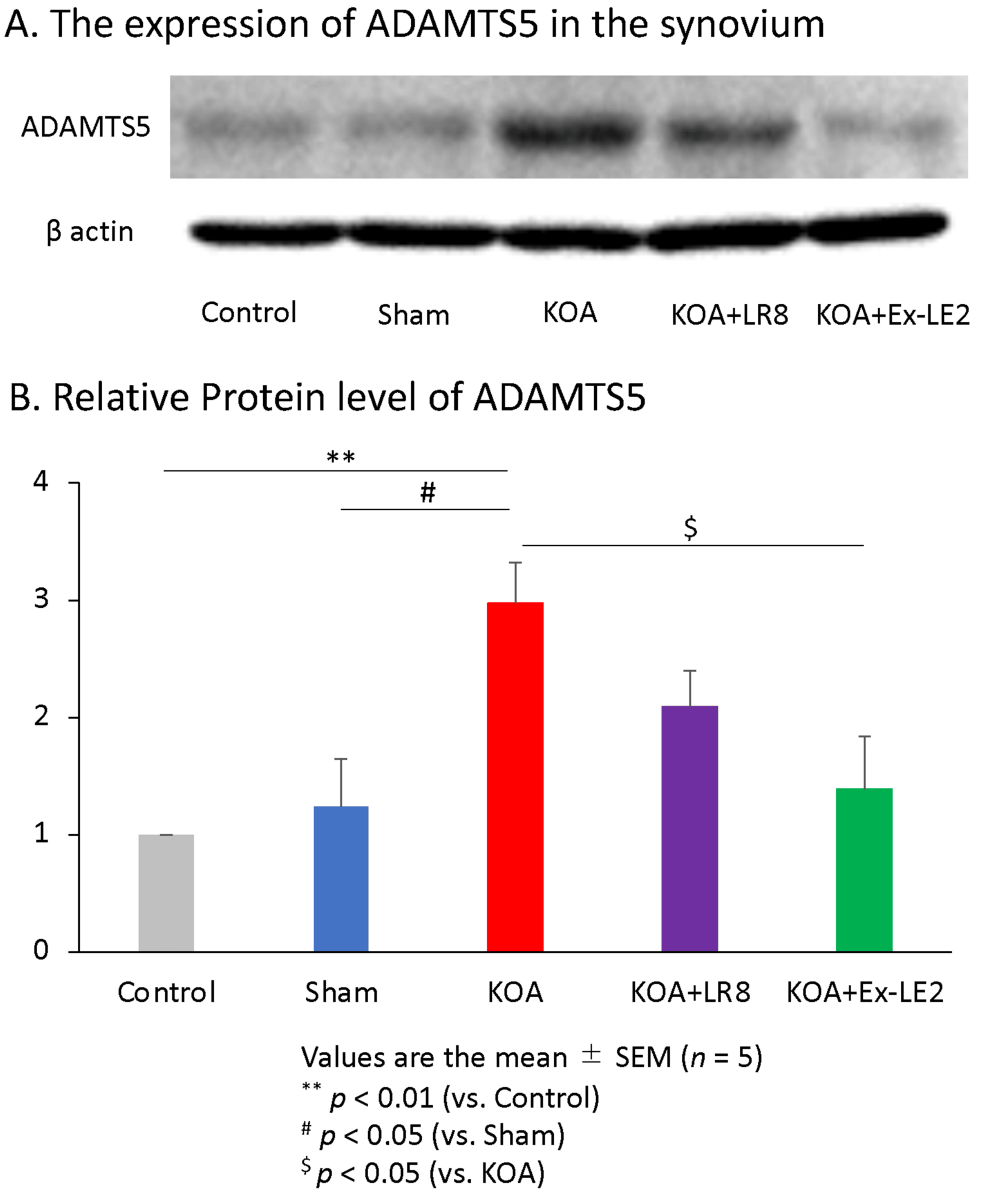
EA at ST36-Ex-LE2 reduced ADAMTS5 expression in the synovial tissue. Electroacupuncture at ST36 and Ex-LE2 inhibited the expression of ADAMTS5 in the synovium in the KOA rat model. (A) The expression of ADAMTS5 in each group was analyzed by Western blot on day 28 post-surgery. (B) Quantification of immunoblots. All data represent mean ± SEM (n = 5). ^**^ P < 0.01 versus the control group, ^#^ P < 0.05 versus the sham group, ^$^ P < 0.05 versus the KOA group based on the ANOVA followed by the Tukey's test. ADAMTS5, A Disintegrin and Metalloproteinase with Thrombospondin motifs 5; KOA, Knee osteoarthritis; ST36, Zusanli; LR8, Ququan; Ex-LE2, Heding

The onset of KOA begins with the degradation of aggrecan in articular cartilage. Therefore, the expression levels of ADAMTS5 in synovial tissue from each group were measured four weeks after surgery using Western blotting. The Western blot analysis revealed that DMM operation increased ADAMTS5 expression in knee synovial tissues (control group: F(4,20) = 5.51, p < 0.01; sham group: F(4,20) = 5.51, p < 0.05), which was inhibited by EA at ST36-Ex-LE2 (F(4,20) = 5.51, p < 0.05) but not at ST36-LR8 (F(4,20) = 5.51, p < 0.05) (Figure 6). On the other hand, there was no significant difference between the ST36-LR8 group and the control (F(4,25) = 6.49, p < 0.18) or sham (F(4,25) = 6.49, p = 0.36) groups. These results suggest that EA at ST36-LR8 has the effect of suppressing ADAMTS5 expression in the synovium, but the effect is not as strong as that of ST36-Ex-LE2 (Figure 6). The changes in synovial ADAMTS5 expression and OARSI score in each group were parallel (Figure 6). These data indicate that EA at ST36-Ex-LE2 inhibits the degeneration of articular cartilage by suppressing the expression of ADAMTS5 in the knee synovium.

## Discussion

In the KOA model rats, decreased motor coordination, increased pain, degeneration of articular cartilage, and increased expression of ADAMTS5 were observed, but these were improved by EA. Regarding the difference in the effect of acupoints, EA at ST36-Ex-LE2 significantly inhibited cartilage degeneration and increased expression of ADAMTS5 in the KOA group. In this experiment, the rats in the KOA group showed decreased motor coordination and increased expression of *c-fos* in the dorsal horn of the spinal cord compared to the control group, but these symptoms were significantly inhibited by EA at ST36-LR8 and ST36-Ex-LE2.

The rotarod test is a test to evaluate motor coordination, including the effect of pain on movement [21]. It has been shown that allodynia occurs in DMM rats four weeks after surgery [22]. Lee et al. also reported that pain induced by KOA leads to a decrease in the animals' hind limb grip strength [23]. Therefore, the reduced time spent on the rod by rats in the KOA group in this experiment may be partly due to knee joint pain, and the improved performance in the rotarod test in the EA group may be related to the pain relief caused by EA.

We previously reported that phosphorylation-related extracellular signal-regulated kinase (pERK), a marker of central sensitization that enhances the excitability of sensory neurons and causes allodynia, was increased in the spinal dorsal horn of DMM model rats four weeks after surgery [18]. Furthermore, it has been shown that *c-fos* expression is increased downstream of ERK activation in the ERK signaling pathway [24]. Thus, pERK and *c-fos* expression play an important role in the development of allodynia [24]. EA exerts analgesic effects via endogenous opioids [9]. It has also been reported that the low-frequency EA used in this experiment suppresses the increase in *c-fos* expression in the spinal dorsal horn induced by noxious stimulation via the μ-opioid receptor (MOR) [9]. Recent studies have also shown that acupuncture stimulation of mast cells, which are abundant at acupoints [20,25], activates these mast cells through the TRPV2 channel and promotes the release of adenosine [26]. Adenosine acts on adenosine A1 receptors in the peripheral nerves that innervate acupoints, ultimately exerting an analgesic effect [26,27]. Considering these reports, it is possible that EA inhibits pain and *c-fos* expression through multiple mechanisms in this experiment.

In this experiment, the protective effect of EA of ST36-Ex-LE2 on articular cartilage was confirmed by Safranin O-Fast Green staining and the OARSI score. The expression of ADAMTS5 in synovial tissue was also suppressed by the EA of ST36-Ex-LE2. In KOA, synovial fibroblasts activated by DAMPs produce inflammatory mediators, aggrecanases including ADAMTS5, and matrix metalloproteinases, which lead to the development of KOA [7]. These released substances further damage the joints and promote the progression of KOA. This vicious cycle of joint destruction was interrupted by EA at ST36-Ex-LE2. Interestingly, the expression level of ADAMTS5 does not seem to be regulated by IL-1β or TNFα [1].

Mast cells are abundant in the skin and subcutaneous tissues of ST36 [25] and Ex-LE2 [20], and acupuncture treatment has been shown to increase adenosine release [26,27]. Ex-LE2 is anatomically aligned with the suprapatellar capsule, which is the most common site of KOA synovitis [20]. Since mast cells are increased in inflamed synovial tissue, Ex-LE2 in KOA can be considered to be a mast cell-rich acupoint within the tissue between the skin and the synovium. In addition, it has been reported that under inflammation conditions, synovial fibroblasts express adenosine 2A (A2A) and 3 (A3) receptors and that binding of adenosine to these receptors inhibits the activation of synovial fibroblasts [28,29]. Furthermore, it has been shown that intra-articularly administered A2A receptor agonists and orally administered A3 receptor agonists prevent the development of KOA [28,29]. These findings suggest that EA at Ex-LE2 may promote the release of adenosine from mast cells in the skin, subcutaneous tissue, and synovium, suppress the expression of ADAMTS5 in synovial fibroblasts, and prevent the degeneration of articular cartilage.

As mentioned above, mast cells are abundantly distributed in ST36 and Ex-LE2 [20,25,28,29]. In contrast, no reports indicate a high mast cell distribution at LR8. In the rotarod test, EA at ST36-Ex-LE2 significantly prolonged the time spent on the rod on days 14, 21, and 28 after surgery compared with the KOA group. However, EA at ST36-LR8 was able to prolong the time spent on the rod on days 14 and 28 but not on day 21 after surgery compared with the KOA group. In the ST36-Ex-LE2 pair, each acupoint is rich in mast cells, whereas in the ST36-LR8 pair, only ST36 is a mast cell-rich acupoint. The reason for these different results may be related to the number of acupoints that are enriched in mast cells. In addition, EA at ST36-Ex-LE2 suppressed cartilage degeneration and ADAMTS5 expression, whereas EA at ST36-LR8 did not. The reason for these different results may also depend on whether Ex-LE2, an acupoint rich in mast cells in the synovium of KOA, is included in the target of treatment. It is possible that EA stimulation of Ex-LE2 promoted adenosine release from mast cells, inhibiting the activation of synovial fibroblasts, thereby suppressing ADAMTS5 expression. Finally, considering the characteristics of these three acupoints, EA at ST36-LR8 may not have shown a stronger inhibitory effect on the degeneration of articular cartilage than ST36-Ex-LE2.

Currently, there are no DMOADs that inhibit the degeneration of articular cartilage in KOA, and the main goal of KOA treatment is to suppress pain and restore function. Although ADAMTS5 is a target for OA treatment, inhibition or deficiency of ADAMTS5 induces cardiovascular side effects [30]. Furthermore, it has been pointed out that the administration of existing NSAIDs and steroids increases the risk of gastrointestinal and cardiovascular diseases and loss of cartilage volume [3]. In this situation, EA has the potential to become an alternative to DMOADs for the treatment of KOA. It has been reported that an average of 50% of patients who underwent meniscectomy after meniscal injury will develop KOA within 20 years [31]. Therefore, early EA intervention in young athletes with meniscal injuries may be effective in preventing KOA. In addition, the results of this study revealed that, among multiple acupoints, the combination of ST36-Ex-LE2 is effective for both pain and articular cartilage degeneration. This provides a clinically useful guide for the acupoints to be selected to prevent KOA after acute meniscal injury. Furthermore, the fact that the extra acupoint Ex-LE2, which has been considered to have a specific effect on knee diseases, has a unique effect and mechanism on KOA will undoubtedly be a breakthrough in advancing future research on other extra acupoints. Based on these results, in the future, epidemiological studies will be necessary to investigate the effects and mechanisms of early intervention with EA using different acupoints on the onset and progression of KOA in humans after meniscus injury. It is also important to clarify the conditions for effective EA for KOA, such as the causes that induce KOA for which EA is applicable, the appropriate timing of intervention, the selection of the acupoints with the most therapeutic effect, and the frequency of treatment, in order to increase the versatility of EA.

One limitation of this study is that the detailed mechanism by which EA suppresses ADAMTS5 expression has not been elucidated. Specifically, the actual number of mast cells and the concentration of degranulated adenosine at the three acupoints used in this experiment have not been clarified. In addition, the expression levels of A2A and A3 receptors in synovial fibroblasts at Ex-LE2 need to be verified. Recently, it has been suggested that the activation of synovial fibroblasts leading to the progression of KOA is related to the NF-kB signaling pathway, which plays a central role in immune responses [32]. NK-kB activation in synovial fibroblasts cannot be suppressed by IL-1β and TNFα antagonists [33]. Future studies need to determine whether EA-induced adenosine binding to A2A and A3 receptors inhibits NF-κB activation in synovial fibroblasts.

In this experiment, EA applied to ST36-Ex-LE2 induced analgesic effects in KOA and also inhibited the increase in ADAMTS5 expression in the synovium, preventing articular cartilage degeneration. On the other hand, EA applied to ST36-LR8 showed analgesic effects in KOA, but the increase in ADAMTS5 expression in the synovium and the inhibitory effects on articular cartilage degeneration were not complete. The finding that different effects can be induced even at acupoints located around the knee joint is very interesting, and future research to clarify the detailed mechanisms will be important. Additionally, studies are needed to clarify the causal relationship between EA-induced inhibition of cartilage degeneration and pain relief.

## Conclusions

This study investigated the effects of EA at different acupoints on KOA in a rat model, focusing on its influence on pain relief and cartilage degeneration, particularly through the regulation of ADAMTS5 expression. The results demonstrated that EA applied to the acupoint pair ST36-Ex-LE2 significantly inhibited the degeneration of articular cartilage and reduced the expression of ADAMTS5, which is a key enzyme in cartilage breakdown. In contrast, EA applied to the ST36-LR8 pair was less effective in preventing cartilage degeneration, although both acupoint combinations showed a similar reduction in pain, as evidenced by decreased *c-fos* expression and improved mobility in the rotarod test.

This suggests that the selection of specific acupoints, particularly those with unique effects such as Ex-LE2, could be crucial in the management of KOA. The findings highlight EA's potential as an alternative therapy for KOA, offering benefits in both pain management and cartilage preservation, possibly positioning it as a future non-pharmacological treatment for osteoarthritis. In order to realize this, well-designed clinical trials are essential.
